# The Antidepressant-like Activity, Effects on Recognition Memory Deficits, Bioavailability, and Safety after Chronic Administration of New Dual-Acting Small Compounds Targeting Neuropsychiatric Symptoms in Dementia

**DOI:** 10.3390/ijms231911452

**Published:** 2022-09-28

**Authors:** Magdalena Jastrzębska-Więsek, Magdalena Kotańska, Aleksandra Grzeszczak, Anna Jaromin, Maria Walczak, Anna Partyka, Joanna Gdula-Argasińska, Magdalena Smolik, Agnieszka Zagórska

**Affiliations:** 1 Department of Clinical Pharmacy, Jagiellonian University Medical College, Medyczna 9, 30-688 Cracow, Poland; 2 Department of Pharmacological Screening, Jagiellonian University Medical College, Medyczna 9, 30-688 Cracow, Poland; 3 Department of Lipids and Liposomes, Faculty of Biotechnology, University of Wrocław, 50-383 Wrocław, Poland; 4 Chair and Department of Toxicology, Jagiellonian University Medical College, Medyczna 9, 30-688 Cracow, Poland; 5 Department of Radioligands, Jagiellonian University Medical College, Medyczna 9, 30-688 Cracow, Poland; 6 Department of Medicinal Chemistry, Jagiellonian University Medical College, Medyczna 9, 30-688 Cracow, Poland

**Keywords:** drug-like compounds, safety pharmacology, cytotoxicity, dementia, multitarget compounds

## Abstract

This study aimed to extend the body of preclinical research on prototype dual-acting compounds combining the pharmacophores relevant for inhibiting cyclic nucleotide phosphodiesterase 10 (PDE10A) and serotonin 5-HT_1A_/5-HT_7_ receptor (5-HT_1A_R/5-HT_7_R) activity into a single chemical entity (compounds PQA-AZ4 and PQA-AZ6). After i.v. administration of PQA-AZ4 and PQA-AZ6 to rats, the brain to plasma ratio was 0.9 and 8.60, respectively. After i.g. administration, the brain to plasma ratio was 5.7 and 5.3, respectively. An antidepressant-like effect was observed for PQA-AZ6 in the forced swim test, after chronic 21-day treatment via i.p. administration with 1 mg/kg/day. Both compounds revealed an increased level of brain-derived neurotrophic factor (*Bdnf)* mRNA in the hippocampus and prefrontal cortex. Moreover, PQA-AZ4 and PQA-AZ6 completely reversed (+)-MK801-induced memory disturbances comparable with the potent PDE10 inhibitor, compound PQ-10. In the safety profile that included measurements of plasma glucose, triglyceride, and total cholesterol concentration, liver enzyme activity, the total antioxidant activity of serum, together with weight gain, compounds exhibited no significant activity. However, the studied compounds had different effects on human normal fibroblast cells as revealed in in vitro assay. The pharmacokinetic and biochemical results support the notion that these novel dual-acting compounds might offer a promising therapeutic tool in CNS-related disorders.

## 1. Introduction

The potency of a small molecule to become a drug is generally described as drug-likeness. Good solubility, membrane permeability, long half-life, and a pharmacophore pattern to interact specifically with a target protein are basic drug-likeness characteristics [1]. In the drug discovery process, the chance for a molecule to become an oral drug is established at an early step from structural or physicochemical inspections [2], even though a highly potent compound against a drug target with optimal physicochemical properties may not be efficacious in in vivo studies based on poor pharmacokinetic properties. Additionally, in early drug discovery, the selection of promising lead compounds requires a profile of cytotoxicity and the effects of the test substance on vital functions after repeated dose studies. The problem becomes extremely important in multitarget drug discovery, where compounds comprise activity related to more than a single biological target [3,4]. Multitarget compounds trigger novel biochemical pathways and the delayed consequences of biochemical changes must be carefully examined, particularly when they are multifactorial. The systematic search for rational target combinations, innovations in computational technologies, crystallography, fragment-based design, and recent in vitro pharmacological methods have added to the potential of multitarget compound development. Currently, multifactorial diseases or diseases where the development of therapeutic resistance is observed (e.g., neurodegenerative, and psychiatric disorders or infectious diseases, cancer, and metabolic/cardiovascular diseases) are the focus of multitarget drug discovery [5,6,7,8,9]. Lastly, we designed and synthesized a library of dual-acting compounds as part of the search for well-tolerated and effective therapies for neuropsychiatric symptoms (NPS), such as psychosis, depression, and anxiety in dementia patients. The strategy was based on designing structures of prototype dual-acting compounds combining the pharmacophores relevant for inhibiting cyclic nucleotide phosphodiesterase 10 (PDE10A) and serotonin 5-HT_1A_/5-HT_7_ receptor (5-HT_1A_R/5-HT_7_R) activity into a single chemical entity. Computer-aided molecule techniques made it possible to design these drug-like compounds. The most potent molecules, PQA-AZ4 and PQA-AZ6 derivatives of 6,7-dimethoxy-quinazoline, were 5-HT_1A_R/5-HT_7_R ligands (HT_1A_R K_i_ 18.0 ± 1.8 nM and 346.0 ± 20.2 nM, respectively; 5-HT_7_R K_i_ 234.0 ± 15.0 nM and 136.0 ± 15.0 nM, respectively), which acted as partial agonists of 5-HT_1A_R and exhibited inhibitory activity of PDE10A (IC_50_ = 7.07 µM and 5.68 µM, respectively) (Table 1) [10]. In preliminary pharmacological evaluation, after acute treatment in vivo, compounds displayed antipsychotic and antidepressant-like activity, and restored recognition memory deficits in mice [10]. Moreover, the preliminary pharmacokinetic screening revealed that the lead compounds penetrated the blood–brain barrier [10].

**Scheme 1:**
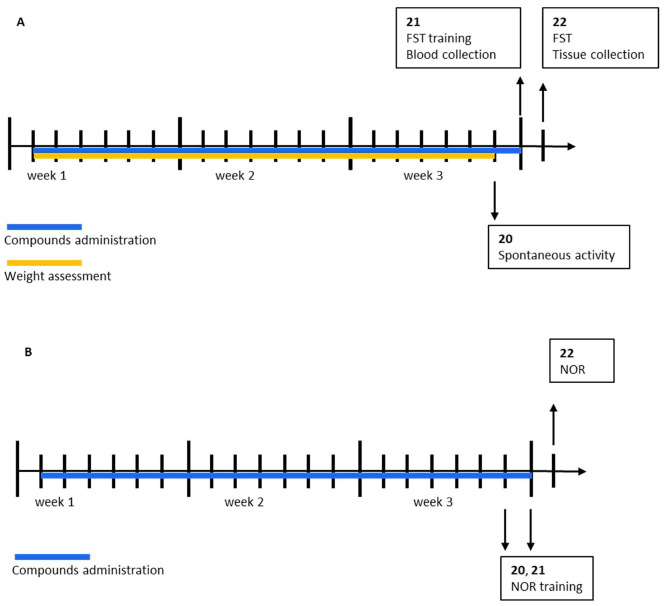
Schematic diagrams of experimental procedures. (**A**) Chronic treatment for FST and ex vivo studies (PQA-AZ4, PQA-AZ6, and PQ-10, i.p., 1 mg/kg/day). (**B**) Chronic treatment for NOR test (i.p. PQA-AZ4—1 mg/kg/day; PQA-AZ6—3 mg/kg/day, PQ-10—0.3 mg/kg/day).

The goal of the present study was to extend the body of preclinical research on PQA-AZ4 and PQA-AZ6 (Scheme 1). The pharmacokinetic profile was extended to the bioavailability measurement after intravenous (i.v.) and intragastric (i.g.) administration of PQA-AZ4 and PQA-AZ6 in rats. Next, the potential antidepressant-like activity and/or the ability to restore recognition memory deficits of the compounds after their chronic (21 days) intraperitoneal (i.p.) administration to rats were assessed. To assess the potential side effects of PQA-AZ4 and PQA-AZ6 after chronic i.p. administration, the impact of the investigated compounds on metabolic parameters such as plasma glucose, triglyceride, total cholesterol concentration, and the total antioxidant activity of serum, together with weight gain was determined. Moreover, the liver enzyme activity after chronic i.p. administration was assessed. Finally, the potential cytotoxic effect of PQA-AZ4 and PQA-AZ6 was determined.

## 2. Results

### 2.1. Bioavailability of PQA-AZ4 and PQA-AZ6 in Rats

Bioavailability was assessed by determining the area under the plasma concentration–time curve (AUC). The detailed plots of mean plasma and brain concentrations versus time profile for PQA-AZ4 and PQA-AZ6 after i.v. and i.g. administration are presented in the Supplementary Materials (Figures S1–S8, respectively). The pharmacokinetic parameters, calculated by the noncompartmental approach, are given in Table 2 and Table 3.

### 2.2. Antidepressant-like Activity of Repeated Administration of PQA-AZ4, PQA-AZ6 in the Forced Swim Test (FST) in Rats

The acute i.p. administration of compounds PQA-AZ4 and PQA-AZ6 at a dose of 1 mg/kg did not change the immobility of the rats, while the reference compound PQ-10 (1 mg/kg i.p.) showed potential antidepressant-like properties (details are given in the Supplementary Materials, Table S1). Therefore, we decided to check the activity of these compounds in the forced swim test (FST) after their chronic administration to rats for 21 days. At the end of the FST, the animals were killed, and brain structures (hippocampus and prefrontal cortex) were collected to assess the expression of brain-derived neurotrophic factor (BDNF) using the quantitative reverse transcription-polymerase chain reaction (qPCR-RT) method.

**Figure 1:**
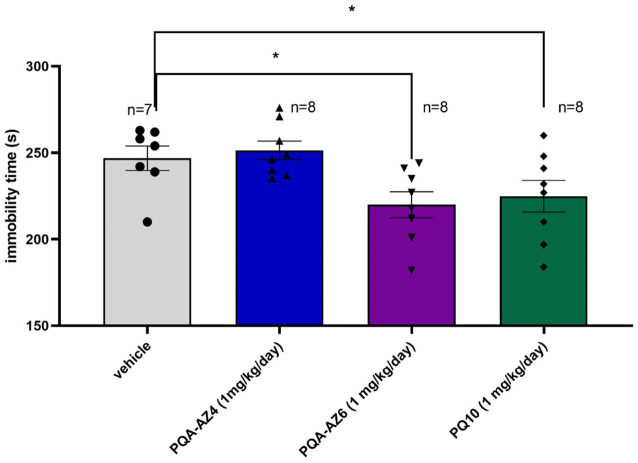
Effects of repeated administration of PQA-AZ4, PQA-AZ6, and PQ-10 on the duration of immobility in the FST in rats. PQA-AZ4, PQA-AZ6, and PQ-10 were administered i.p. once daily for 21 consecutive days. Data represent the mean ± SEM, n = 7–8 rats per group; one-way ANOVA followed by Bonferroni’s post-hoc test, * *p* < 0.05 vs. respective vehicle-treated group.

Only compound PQA-AZ6 and PQ-10 showed antidepressant-like properties in the FST after their chronic i.p. administration to rats. PQA-AZ6 decreased immobility by about 11% (one-way ANOVA F(1,13) = 6.6392; *p* < 0.05) vs. control group; similar to reference compound PQ-10, which decreased immobility by about 9% (one-way ANOVA F(1,13) = 5.0743; *p* < 0.05) vs. control rats. Compound PQA-AZ4 was inactive in this test after its repeated administration at the dose of 1 mg/kg/day for 21 days (Figure 1; one-way ANOVA F(1,13) = 0.2627; NS).

**Figure 2:**
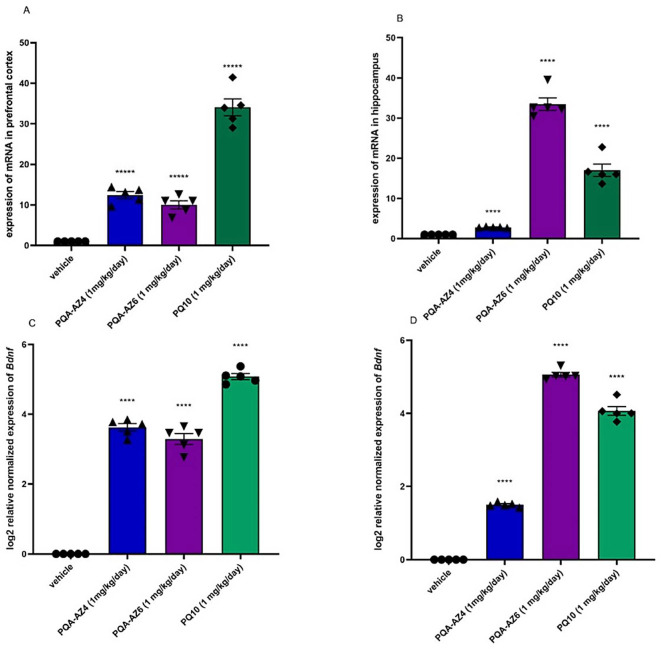
Effects of repeated administration of PQA-AZ4, PQA-AZ6, and PQ-10 on expression and relative normalized expression of the *Bdnf* gene in the prefrontal cortex ((**A**,**C**), respectively) and the hippocampus ((**B**,**D**), respectively) in rats. PQA-AZ4, PQA-AZ6, and PQ-10 were administered i.p. once daily to male rats for 21 consecutive days and the tissues were collected immediately after the FST. Data represent the mean ± SD, n = 5 rats per group; statistical analysis was performed using the Student’s *t*-test; **** *p <* 0.0001, ***** *p* < 0.00001 vs. respective vehicle-treated group.

Chronic i.p. administration of PQ-10 at a dose of 1 mg/kg/day for 21 days resulted in the highest statistical expression of *Bdnf* in the prefrontal cortex (PFX) of rats (by about 34-fold, Student’s *t*-test t = −15.7492, df = 8, *p* = 0.0000) (Figure 2A,C). After chronic administration of PQA-AZ4 and PQA-AZ6, the BDNF gene was also upregulated, compared to the control group, in the investigated brain structures (by about 10- and 12-fold, respectively, for PQA-AZ4, Student’s *t*-test t = −13.2270, df = 8, *p* = 0.0001, for PQA-AZ6, Student’s *t*-test t = −8.897441, df = 8, *p* = 0.00002), but the activity was lower than after PQ-10 administration (Figure 2A). Analyzing *Bdnf* gene expression in the hippocampus (Hipp) the highest, and statistically significant, expression of the *Bdnf* gene was observed after chronic i.p. administration of PQA-AZ6 at a dose of 1 mg/kg/day (by about 33-fold, Student’s *t*-test t = −20.8059, df = 8, *p* = 0.0000), while the treatment with compound PQA-AZ4 in the same scheme resulted in lower upregulation of *Bdnf* expression (by about 2.8-fold, Student’s *t*-test t = −35.4736, df = 8, *p* = 0.0000). The reference compound PQ-10 injected i.p. chronically (1 mg/kg/day) also upregulated *Bdnf* expression in the hippocampus (by about 17-fold, Student’s *t*-test t = −10.4826, df = 8, *p* = 0.00006) (Figure 2B,D).

While the obtained results show a relationship between *Bdnf* gene expression and the antidepressant-like properties of the tested compounds, it is not possible to identify the direct connection between the two components.

### 2.3. Memory-Enhancing Properties of Repeated Administration of PQA-AZ4, PQA-AZ6, and PQ-10 in the Novel Object Recognition (NOR) Test in (+)-MK-801 Treated Rats

**Figure 3:**
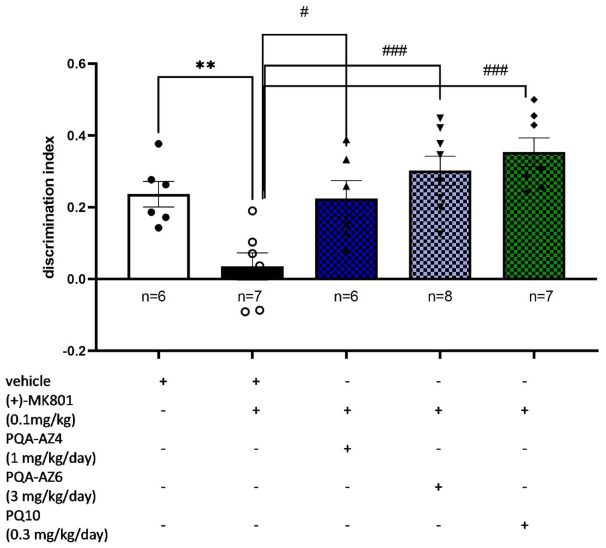
Effects of repeated administration of PQA-AZ4, PQA-AZ6, and PQ-10 on (+)-MK-801-induced memory deficits in the NOR test conducted on rats. PQA-AZ4, PQA-AZ6, and PQ-10 were administered i.p. once daily for 21 consecutive days. (+)-MK-801 was injected i.p. 30 min before the first trial (familiarization, T1) phase. Data represent the mean ± SEM, n = 6–8 rats per group; one-way ANOVA followed by Bonferroni’s post hoc test, ** *p* < 0.01 vs. respective vehicle-treated group # *p* < 0.05, ### *p* < 0.001 vs. respective (+)-MK-801 treated group.

Memory-enhancing properties of the compounds were investigated in the NOR test in rats treated with (+)-MK-801 after repeated administration of the compounds PQA-AZ4, PQA-AZ6, and PQ-10. Minimum active doses obtained in the NOR test after acute treatment with dual-acting PDE10-inhibitors and the serotonin receptor ligands were selected for this experiment. The discrimination index (DI) was used to reflect the preference of rats to explore the novel object rather than the familiar object. The higher value of DI denotes the stronger preference of the rats to explore the new object, reflecting the ability to reverse (+)-MK-801-induced memory impairments. All investigated compounds restored recognition memory deficits induced by (+)-MK-801 administration, after chronic administration of PQA-AZ4 (1 mg/kg/day; one-way ANOVA F(2,16) = 7.818; *p* < 0.01), PQA-AZ6 (3 mg/kg/day; one-way ANOVA F(2,17) = 18.646; *p* < 0.0001), and PQ-10 (0.3 mg/kg/day; one-way ANOVA F(2,18) = 13.326; *p* < 0.001), and the DI value increased significantly compared to control rats treated with (+)-MK-801 alone (Figure 3). The higher value of DI denotes a stronger preference for the novel object that is connected with the ability of the compounds to reverse (+)-MK-801-induced memory impairments and is associated with memory-enhanced properties of the investigated compounds.

All the investigated compounds restored recognition memory deficits induced by (+)-MK-801 administered by i.p. injection, after chronic administration of PQA-AZ4 (1 mg/kg/day i.p.), PQA-AZ6 (3 mg/kg/day i.p.), and PQ-10 (0.3 mg/kg/day i.p.). The DI value increased significantly compared to only the control rats treated with (+)-MK-801 alone (Figure 3).

During the NOR test, the total exploratory time of objects in the recognition phase (T2) was measured after chronic i.p. administration of the investigated compounds to avoid false-positive results in this test that could be connected with the effect of treatment on behavioral parameters, especially locomotor activity. The total exploratory time measured both for novel and familiar objects for these compounds during the T2 trial did not change vs. vehicle- and (+)-MK-801-treated groups (Table 4). Hence, the results obtained in the NOR test seem to be specific for the potential ability of PQA-AZ4, PQA-AZ6, and PQ-10 to reverse memory deficits in rats after their chronic administration to the animals. Moreover, for compound PQA-AZ4, some significant decreases in exploratory activity compared to vehicle- and (+)-MK-801-treated animals were observed while, simultaneously, the time of exploration of the novel object in the T2 phase was increased (Table 4, Figure 3).

### 2.4. Effects of Repeated Administration of PQA-AZ4, PQA-AZ6, and PQ-10 on Spontaneous Locomotor Activity

**Figure 4:**
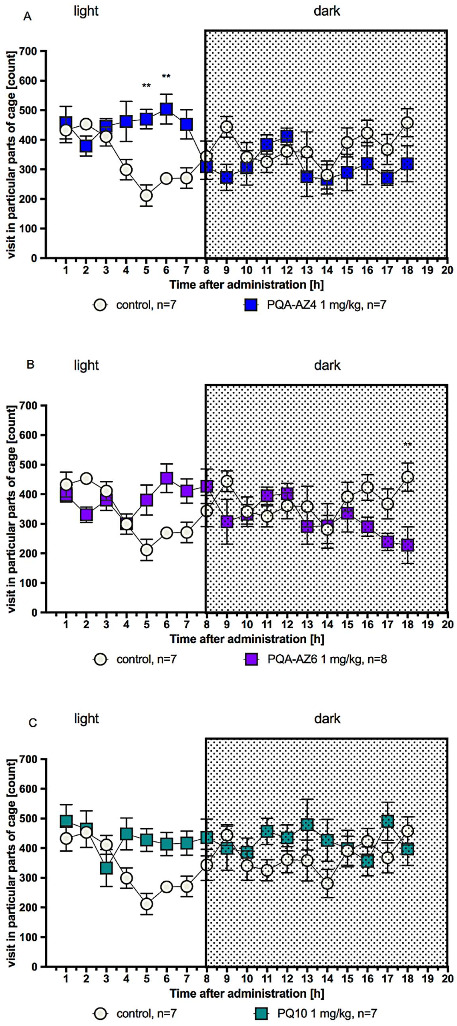
Effects of administration of PQA-AZ4, PQA-AZ6, or PQ10 on the spontaneous activity of rats during light and dark phases: (**A**)—after the 20th administration of PQA-AZ4, (**B**)—after the 20th administration of PQA-AZ6, (**C**)—after the 20th administration of PQ10. Each plot represents the mean ± SEM, n = 7–8 rats per group; two-way ANOVA followed by Bonferroni’s post hoc test, ** *p* < 0.01 vs. respective vehicle-treated group.

The spontaneous activity of rats five and six hours after 20-day administration of PQA-AZ4 (1 mg/kg/day i.p.) was increased vs. respective vehicle-treated groups of rats (two-way ANOVA F(17,204) = 4.35; *p* < 0.001) (Figure 4A). The spontaneous activity of rats eighteen hours after 20-day administration of PQA-AZ6 (1 mg/kg/day i.p.) was decreased vs. respective vehicle-treated groups of rats (two-way ANOVA F(17,221) = 3.405; *p* < 0.0001) (Figure 4B). No significant effect was observed on the spontaneous activity of rats after 20-day administration of PQ10 (1 mg/kg/day i.p.) vs. respective vehicle-treated groups of rats (two-way ANOVA F(17,204) = 1.653; NS) (Figure 4C).

### 2.5. Effects of Repeated Administration of PQA-AZ4 and PQA-AZ6 on Body Weight of Rats

**Figure 5:**
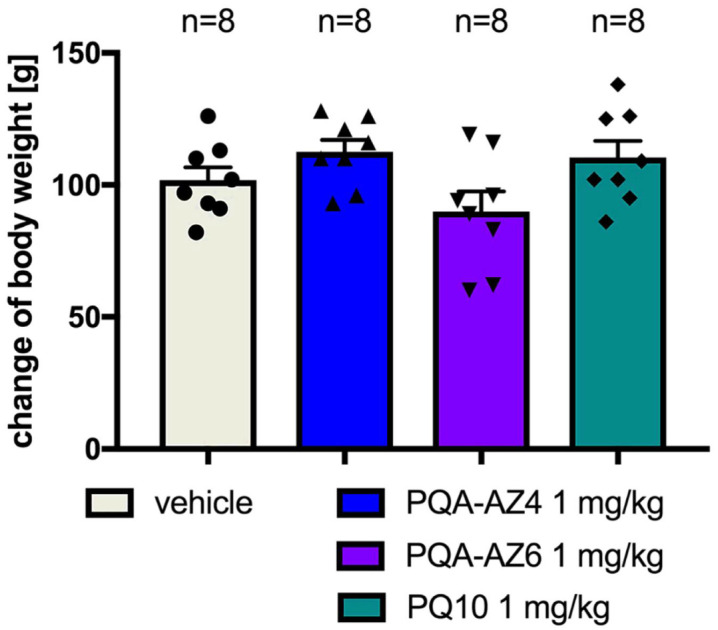
Effects of repeated administration of PQ-10, PQA-AZ4, and PQA-AZ6 on body weight in rats. PQA-AZ4 and PQA-AZ6 were administered i.p. once daily for 21 consecutive days. Data represent the mean ± SEM, n = 8 rats per group; statistical analysis was performed using the Student’s *t*-test.

No significant effect was observed on the weight gain of rats after 20-day administration of PQA-AZ4 (Student’s *t*-test t = 1.59, df = 14, *p* = 0.1341) and PQA-AZ6 (Student’s *t*-test t = 1.298, df = 14, *p* = 0.2154) vs. respective vehicle-treated groups of animals. Treatment with reference compound PQ-10 (1 mg/kg/day i.p.) also did not result in a change in the weight gain of animals (Student’s *t*-test t = 1.079, df = 14, *p* = 0.2990) (Figure 5).

### 2.6. Effects of Repeated Administration of PQA-AZ4, PQA-AZ6, and PQ-10 on Serum Glucose, Triglyceride, and Cholesterol Concentrations in Rats

**Figure 6:**
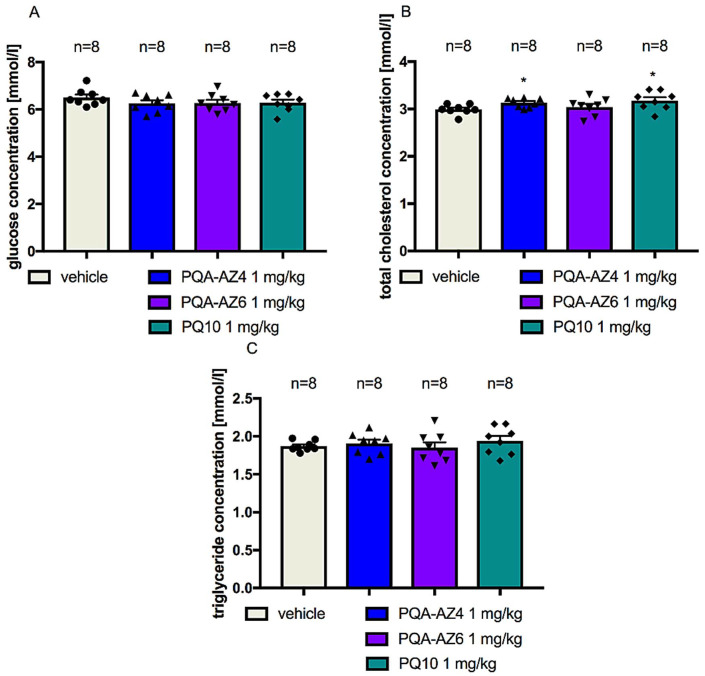
Effects of repeated administration of PQA-AZ4, PQA-AZ6, and PQ-10 on glucose (**A**), triglyceride (**C**), and cholesterol concentration (**B**) in rat serum. PQA-AZ4, PQA-AZ6, and PQ-10 were administered i.p. once daily for 21 consecutive days. Data represent the mean ± SEM, n = 8 rats per group; statistical analysis was performed using the Student’s *t*-test, * *p* < 0.05 vs. respective vehicle-treated group.

The repeated administration of the investigated compounds, namely, PQA-AZ4 and PQA-AZ6, and reference compound (PQ-10), at a dose of 1 mg/kg/day i.p. did not significantly change the concentration of glucose (Student’s *t*-test t = 1.338, df = 14, *p* = 0.2023; Student’s *t*-test t = 1.271, df = 14, *p* = 0.2245; Student’s *t*-test t = 1.21, df = 14, *p* = 0.2462, respectively) (Figure 6A) and triglycerides (Student’s *t*-test t = 0.6514, df = 14, *p* = 0.5253; Student’s *t*-test t = 0.2593, df = 14, *p* = 0.7992; Student’s *t*-test t = 1.013, df = 14, *p* = 0.3284, respectively) (Figure 6C), as compared to those measured in vehicle-treated rats. Only a slight but statistically significant (*p* < 0.05) increase in the total cholesterol concentration level was observed for PQA-AZ4 (Student’s *t*-test t = 2.752, df = 14, *p* = 0.0156) and PQ-10 (Student’s *t*-test t = 2.343, df = 14, *p* = 0.0344) vs. respective vehicle-treated groups. These results are not clinically significant (Figure 6B).

### 2.7. Effects of Repeated Administration of PQA-AZ4, PQA-AZ6, and PQ-10 Activities of Alanine Aminotransferase (AlaT), Aspartate Aminotransferase (AspaT), Gamma-Glutamyltransferase (γGTP), and Alkaline Phosphatase (ALP) in Rat Serum

**Figure 7:**
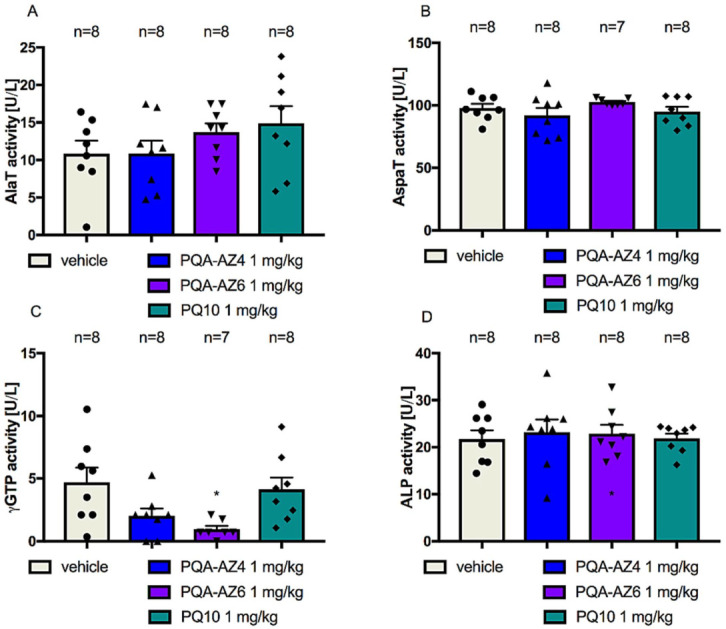
Effects of repeated administration of PQA-AZ4, PQA-AZ6, and PQ-10 on activities of enzymes: alanine aminotransferase (AlaT, (**A**)), aspartate aminotransferase (AspaT, (**B**)), gamma-glutamyltransferase (γGTP, (**C**)), and alkaline phosphatase (ALP, (**D**)) in serum. PQA-AZ4, PQA-AZ6, and PQ-10 were administered i.p. once daily for 21 consecutive days. Data represent the mean ± SEM, n = 7–8 rats per group; statistical analysis was performed using the Student’s *t*-test, * *p* < 0.05 vs. respective vehicle-treated group.

There were no significant changes in the activity of the enzymes tested in serum. The γGTP activity determined after administration of PQ-AZ6 was, statistically, significantly lower (Student’s *t*-test t = 2.906, df = 13, *p* = 0.0123) than that determined in the control group, but this was not clinically significant (Figure 7).

### 2.8. Ferric-Reducing Antioxidant Power after Chronic Administration of PQA-AZ4, PQA-AZ6, and PQ-10 to Rats

**Figure 8:**
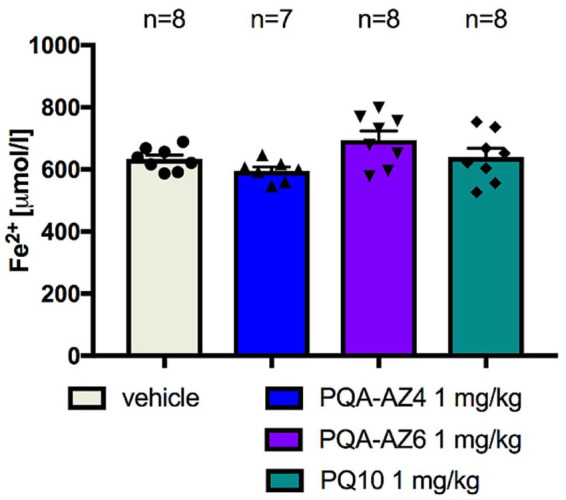
Ferric-reducing antioxidant power in serum after repeated administration of PQA-AZ4, PQA-AZ6, or PQ-10; PQA-AZ4, PQA-AZ6, and PQ-10 were administered i.p. once daily for 21 consecutive days. Data represent the mean ± SEM, n = 7–8 rats per group; statistical analysis was performed using the Student’s *t*-test.

None of the compounds tested had a significant effect on the total antioxidant activity of serum. The amount of iron ions (Fe^2+^) formed was comparable in sera collected from animals treated with tested compounds, namely, PQA-AZ4 and PQA-AZ6 (Student’s *t*-test t = 2.1, df = 13, *p* = 0.0558; Student’s *t*-test t = 1.928, df = 14, *p* = 0.0744, respectively) or reference compound PQ-10 (Student’s *t*-test t = 0.194, df = 14, *p* = 0.8489) with the level of these ions in serum collected from control animals (Figure 8).

### 2.9. Cytotoxic Effects of PQA-AZ4 and PQA-AZ6 on Normal Human Dermal Fibroblast Cells (NHDF)

**Figure 9:**
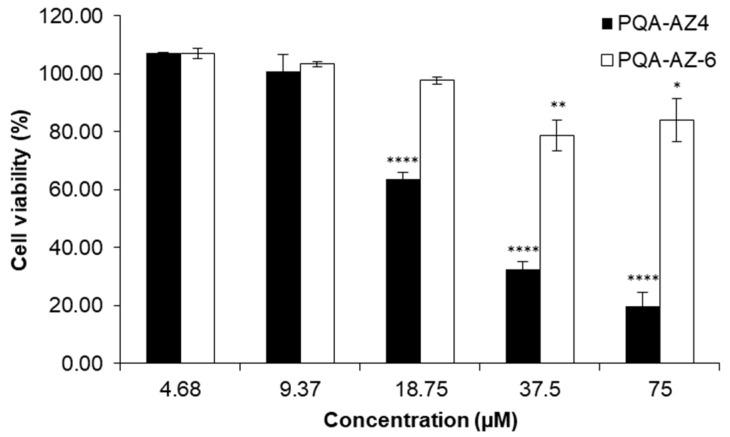
Cytotoxic effects of PQA-AZ4 and PQA-AZ6 on NHDF cells, determined by the MTT assay after 48 h of incubation. Data represent the mean ± SEM. Statistical significance was estimated using one-way ANOVA with the post hoc Dunnett’s test for multiple comparisons and presented as * *p* ≤ 0.05, ** *p* ≤ 0.01, and **** *p* ≤ 0.0001.

The cytotoxic effects of PQA-AZ4 and PQA-AZ6 against normal human dermal fibroblast cells were preliminarily determined by an MTT assay after 48 h of exposure to the compounds. The dose-dependent responses for the NHDF cell-line are shown in Figure 9. Compound PQA-AZ4 was more toxic to these human cells. This was evidenced by the fact that, at the highest tested concentration of PQA-AZ4, cell viability was at the level of 19.93%, while after incubation in the presence of PQA-AZ6 at the same concentration, it was 83.99%.

## 3. Discussion

The nonclinical safety study recommendations to support human clinical trials usually include safety pharmacology studies, repeated dose toxicity studies, toxicokinetic, and nonclinical pharmacokinetic studies [11].

Previously conducted pharmacokinetic studies of compounds PQA-AZ4 and PQA-AZ6 after i.p. administration revealed their high permeability of the brain–blood barrier (BBB), and significant distribution in brain structures such as the frontal cortex, striatum, and hippocampus [10]. Those studies showed that PQA-AZ4 and PQA-AZ6 were distributed to the brain structures where PDE10A splice variants have been identified. In the present study, the comparison of the pharmacokinetic parameters of PQA-AZ4 and PQA-AZ6 after i.g. and i.v. administration was performed. PQA-AZ4 and PQA-AZ6 were eliminated rather rapidly from rats’ bodies, with a half-life of ca. 1 h. The volume of distribution for PQA-AZ6 was higher (ca. 4.7 L/kg), which indicates that the compound is distributed throughout the total body water. After i.g. administration, PQA-AZ4 achieved maximum concentration in the blood within 60 min, whereas, for PQA-AZ6, the maximum concentration was reached after 15 min. Following i.v. administration, PQA-AZ6 more effectively penetrates the blood–brain barrier (brain to plasma ratio of 8.60) compared to PQA-AZ4 (brain to plasma ratio of 0.9). After i.g. administration of PQA-AZ4 and PQA-AZ6, the brain to plasma ratios were similar, namely, 5.7 and 5.3, respectively. The absolute bioavailability of PQA-AZ4 and PQA-AZ6 after i.g. administration was 8.3% and 38.4%, respectively. Collectively, these results suggest that the compounds displayed favorable drug-like pharmacokinetic properties in the rat, which prompted their pharmacodynamic evaluation. Hence, we decided to expand the pharmacodynamic assessment of these compounds.

The 5-HT_1A_ receptor family (5-HT_1A_R) is an attractive target for the pharmacotherapy of pathologies associated with dysfunctional serotonergic neurotransmission, including anxiety, depression, Parkinson’s disease, pain, and schizophrenia. 5HT_1A_R are expressed both as presynaptic autoreceptors in serotonergic cell bodies, in the raphe nuclei (where serotonin is produced), and as postsynaptic heteroreceptors, in multiple regions of the brain, including the cortex, hippocampus, septum, and hypothalamus [12]. Furthermore, pharmacological studies showed that PDE10 inhibitors may be promising drug candidates for the treatment of schizophrenia and neurodegenerative disorders [13,14]. Recently, special attention has been given to multitarget-directed ligands (MTDLs) targeting PDE10 and other enzymes or receptors in the central nervous system (CNS)-related disorders [15,16,17]. Unfortunately, despite the availability of abundant MTDLs affecting PDE10A, until now, no clinical studies have confirmed their efficacy in humans.

Previously conducted pharmacological studies on PQA-AZ4 and PQA-AZ6 revealed that, after acute administration, they demonstrated antipsychotic- and antidepressant-like properties in mice and restored recognition memory deficits in rats [10]. In the work presented, we aimed to expand the preclinical profile of in vivo investigations of PQA-AZ4 and PQA-AZ6. The most interesting question was whether the pharmacological effects would be present after chronic administration of PQA-AZ4 and PQA-AZ6 via the i.p. route over a chronic period of 21 days. The data obtained previously in mice showed antidepressant-like properties for both investigated compounds and for the reference compound PQ-10 (a potent PDE10 inhibitor) after a single administration [10]. Therefore, we first decided to check the antidepressant-like properties of these new compounds in rats after acute administration in the dose range of 0.3–3 mg/kg i.p. The doses were selected as comparable with the doses used previously in the acute NOR test in rats [10]. Considering the previously obtained FST results in mice [10], and the delay in eliciting the therapeutic effect of antidepressant treatment in humans [18], we decided to conduct a 21-day treatment with 1 mg/kg/day of i.p. administration of PQA-AZ4, PQA-AZ6, and PQ-10 in rats. The same dose for all investigated compounds was chosen to compare the pharmacological effects of all three compounds, namely, PQA-AZ4, PQA-AZ6, and PQ-10. Moreover, the same dose was used to measure the effects of repeated administration of PQA-AZ4, PQA-AZ6, and PQ-10 on spontaneous locomotor activity.

The antidepressant-like effect was observed only for PQA-AZ6 and the reference compound PQ-10. It has been proven that antidepressant treatment enhances neuroplasticity and can reverse neuroplasticity deficits that occur during the acute phase of depression [19]. In addition, in acute phase depression, an increase in the number of synaptic monoamines caused by antidepressants is believed to produce secondary neuroplastic transitions that involve transcriptional and translational changes that mediate molecular and cellular plasticity [20]. Furthermore, chronic administration of antidepressant drugs, as well as electroconvulsive therapy, causes upregulation of some neurotrophins, such as brain-derived neurotrophic factor (BDNF), neuronal growth factor (NGF)), and expression of the cAMP response element binding protein (CREB) [20,21,22]. Chronic administration of the investigated compounds revealed an increased level of *Bdnf* mRNA in selected brain structures, namely, the hippocampus (Hipp) and prefrontal cortex (PFX), as determined by quantitative PCR-RT evaluation. However, the highest expression of BDNF mRNA was observed for PQA-AZ6 in the Hipp and PQ-10 in the PFX, while chronic administration of PQA-AZ4 resulted in the lowest expression of *Bdnf* mRNA. These ex vivo biochemical results are in line with the behavioral observation in FST, and the antidepressant-like properties observed with PQA-AZ6 and PQ-10 can be related to the increased BDNF level.

In the next step of our behavioral studies, we investigated the ability of repeated administration of PQA-AZ4, PQA-AZ6, and PQ-10 to reverse (+)MK-801-induced memory disturbances. For the chronic administration of the compounds, the minimal effective doses obtained in acute treatment were chosen [10]. All investigated compounds, namely, PQA-AZ4 (1 mg/kg/day), PQA-AZ6 (3 mg/kg/day), and PQ-10 (0.3 mg/kg/day), restored recognition memory deficits induced by (+)-MK-801 i.p. injection after 21 days of administration, with statistical significance.

The novel object recognition task (NOR test) was used to study the influence of the investigated compounds on visual episodic memory [23]. This test is based on the spontaneous exploration of novel and familiar objects. Pharmacologically induced cognitive deficits in animals are believed to model the impairments seen in humans as, for example, a consequence of developmental cognitive functions, and display a characteristic pharmacological profile, called “cognition enhancers”. Dizocilpine (MK-801) is widely used experimentally as an NMDA receptor antagonist, and (+)MK-801-induced cognitive impairments have been validated as a rodent model related to human cognitive deficits associated with dementia [24] and schizophrenia [25]. (+)MK-801-induced cognitive deficits can be antagonized by putative cognition enhancers. The literature provides sample evidence that cognition enhancers with different modes of action can ameliorate these deficits [24,26,27,28]; PDE10A inhibitors are also among them [13,14,29]. These data support the notion that animal models that demonstrate cognitive deficits induced by the cognition impairer, (+)MK-801, may possess some predictive validity in the search for new compounds with potential impact on memory and learning. (+)MK-801 has been chosen because it induces, by noncompetitive blocking of NMDA receptors [30], cognitive disruptions similar to those associated with dementia [31] and schizophrenia [29,32], which were the potential therapeutic targets for these prototype dual-acting compounds combining the pharmacophores relevant for PDE10A and 5-HT_1A_R/5-HT_7_R activity [10].

Antipsychotic treatment is associated with metabolic disturbance and weight gain effects. However, the association between metabolic change and change in psychopathology is uncertain [33]. Thus, bearing in mind that the PDE10A inhibitors in the dendritic membranes in the medium spiny neurons (MSNs) of the subcortical striatal region of the brain can influence both the indirect and direct dopamine-mediated striatal output pathways [34], we measured the effect of PQA-AZ4 and PQA-AZ6 on weight gain and serum glucose, triglyceride, and cholesterol concentration in rats.

Next, the purpose of the safety pharmacology study was to investigate the effects of the test substances on vital organs. Liver toxicity is one of the most common reasons for terminating development of drugs in humans [35]. Thus, the activities of enzymes such as alanine aminotransferase (AlaT), aspartate aminotransferase (AspaT), gamma-glutamyltransferase (γGTP), and alkaline phosphatase (ALP) were measured after three-week administration of each of the respective compounds in order to check the effect of the tested compounds on the liver cells. Abnormal liver enzyme levels may signal liver damage or alteration in bile flow. The current best practice recommendation for nonclinical safety assessment is that a minimum of four serum parameters should be used to assess hepatocellular and hepatobiliary injury [36,37]. AlaT and AspaT activities assess hepatocellular injury, while γGTP and ALP activity assess hepatobiliary injury [38]. The results showed that no significant changes in the activity of the four tested enzymes were observed after three-week administration of PQA-AZ4 and PQA-AZ6, and that there was also no effect on the total antioxidant activity of serum.

Further, potential cytotoxicity of PQA-AZ4 and PQA-AZ6 was evaluated in vitro on normal human fibroblast cells. Such a cell-based assay is performed to identify the possible potential of studied agents to induce cell toxicity in mammalian cells. Our results indicated that PQA-AZ4 is much more toxic to normal cells than PQA-AZ6. According to ISO 10993-5 [39], a viability above 80% means no cytotoxicity; thus, it can be concluded that PQA-AZ6 is biocompatible and safe in the tested concentration range. However, both compounds are significantly less toxic than the structurally similar compound, a carboxamide derivative of 1H-pyrazolo-[4,3-h]quinazoline, previously described in the literature [40]. Additionally, due to the different models in in vitro and in vivo studies and the concentrations used, it is difficult to compare the results.

Based on the present findings, we conclude that PQA-AZ4 and PQA-AZ6 completely reversed (+)-MK801-induced memory disturbances in rats, comparable with reference compound, PQ-10, while only PQA-AZ6 and PQ-10 revealed antidepressant-like properties in the FST after their 21 days of administration in rats. The pharmacokinetic and biochemical results obtained support the notion that these novel prototype dual-acting compounds combining the pharmacophores relevant for PDE10A and 5-HT_1A_R/5-HT_7_R activity might offer a promising therapeutic tool in CNS-connected disorders, especially for NPS in dementia patients. Future investigations are warranted to assess the mechanism of action of these compounds.

## 4. Materials and Methods

### 4.1. Animals

The behavioral experiments were performed on male Wistar rats: weighing 250–280 g, n = 80 for pharmacokinetic studies, and weighing 190–200 g at the beginning of the chronic treatment (forced swim test, novel object recognition test, locomotor activity, and ex vivo studies), n = 72. The animals were purchased from the Animal House at the Faculty of Pharmacy, Jagiellonian University Medical College, Cracow, Poland. The animals were kept in an environmentally controlled room (temperature of 22 ± 2 °C, humidity 55 ± 10%) on 12 h light/dark cycles (light on at 7:00 AM and off at 7:00 PM) and had free access to food (standard laboratory pellets) and tap water. The rats were housed in groups of 4 in plastic cages (L × W × H) 382 × 220 × 180 mm.

All experiments were conducted in the light phase between 9 AM and 2 PM. Experimental groups consisted of 6–8 randomly selected animals, with detailed information included in captions under each table and figure. Rats were injected with the investigated compounds for 21 days; afterward, behavioral tests were conducted and tissues collected, as required. All the experimental procedures with animals were conducted following EU Directive 2010/63/EU and were approved by the Local Ethics Commission for Animal Experiments of Jagiellonian University in Cracow (Approval No. 1/2017 and 158/2017). The research program complies with the commonly accepted ”3Rs” (replacement and reduction of animals, refinement of experimental conditions and procedures to minimize the harm to animals).

### 4.2. Synthesized Compounds

The compounds investigated, namely, PQA-AZ4 and PQA-AZ6 (UPLC/MS purity > 95%), were synthesized at the Department of Medicinal Chemistry, Jagiellonian University Medical College (synthesis described in [10]); PQ-10, (6,7-dimethoxy-4-[(3R)-3-(2-quinoxalinyloxy)-1-pyrrolidinyl]-quinazoline, was obtained from Sigma-Aldrich (St. Louis, MO, USA). For the studies, the compounds were suspended in 1% aqueous solution of Tween 80, while (+)-MK-801 (hydrogen maleate, Sigma-Aldrich, St. Louis, MO, USA) was dissolved in distilled water. In the behavioral studies, PQA-AZ4, PQA-AZ6, and PQ-10 were injected intraperitoneally (i.p.) once a day (between 9:00 AM and 10:00 AM) for 21 consecutive days at the minimum active doses selected previously, based on acute treatment and assessment in behavioral tests [38] at a volume of 2 mL/kg body weight. The control groups received 1% aqueous solution of Tween 80 (Sigma-Aldrich, St. Louis, MO, USA) in the same schedule. For the pharmacokinetic study, PQA-AZ4 and PQA-AZ6 dissolved in 20% captizol (Ligand, San Diego, CA, USA) were administered intravenously (i.v.) and intragastrically (i.g.) at a dose of 0.5 and 2 mg/kg, respectively, while ketamine (50 mg/kg, (hydrochloride, Biowet Puławy, Puławy, Poland)) and xylazine (8 mg/kg, hydrochloride, Biowet Puławy, Poland)) were injected i.p.

### 4.3. LC/MS/MS Analysis and Pharmacokinetic Studies

HPLC grade methanol, acetonitrile and reagent grade formic acid, hydrochloric acid, potassium dihydrogen phosphate, orthophosphoric acid, and sodium chloride were purchased from Merck (Darmstadt, Germany). Tissues (control blood and brain) were obtained from adult male Wistar rats anesthetized by i.p. injection of 50 mg/kg ketamine plus 8 mg/kg xylazine, and blood samples were collected in heparinized tubes from the heart. Blood samples were collected at 0 (predose), 15, 60, 240, and 1440 min after compound administration. The plasma was separated by centrifugation (1000× *g*, 10 min). Plasma and brain were stored at −80 °C pending analysis. The LC/ESI-MS/MS experiments were performed on a UPLC ACQUITY H-Class PLUS/Xevo TQD (Waters Corporation, Milford, MA, USA) triple-quadrupole mass spectrometer equipped with an electrospray (ESI) ionization interface. Data acquisition and processing were accomplished using MassLynx data collection and integration software. For the chromatographic and mass spectrometric conditions, and sample pretreatment, please see the Supplementary Materials.

Pharmacokinetic parameters were calculated by a noncompartmental approach from the average concentration values, using Phoenix WinNonlin software (Certara, Princeton, NJ 08540 USA). The first-order elimination rate constant (λz) was calculated by linear regression of log concentration versus time. The area under the mean plasma and brain concentration versus time curve (AUC_0__→t_) was calculated from zero to the last concentration point using the linear trapezoidal rule:

(1) $${\mathrm{AUC}}_{0\to t}=\sum_{i=1}^{n}\frac{C_{i}+C_{i+1}}{2}\cdot \left(t_{i+1}-t_{i}\right)$$

where $C_{i}$ is the concentration of the compound.

The area under the first-moment curve (AUMC_0__→t_) was estimated by calculation of the total area under the first-moment curve:

(2) $${\mathrm{AUMC}}_{0\to t}=\sum_{i=1}^{n}\left((t_{i}\cdot C_{i}+t_{i+1}\cdot C_{i+1})/2\right)\cdot \left(t_{i+1}-t_{i}\right)$$

where $t_{i}$ is the time of the last sampling.

Mean residence time (MRT) was calculated as:

(3) $$\mathrm{MRT}=\frac{{\mathrm{AUMC}}_{0\to t}}{{\mathrm{AUC}}_{0\to t}}$$

Systemic clearance (Cl) was calculated as:

(4) $$\mathrm{Cl}=\frac{D_{\mathrm{iv}}}{{\mathrm{AUC}}_{0\to t}}$$

The volume of distribution at steady state (V_ss_) was calculated as:

(5) $$V_{\mathrm{ss}}=\frac{D_{\mathrm{iv}}\cdot {\mathrm{AUMC}}_{0\to t}}{{\left({\mathrm{AUC}}_{0\to t\;}\right)}^{2}}$$

The absolute bioavailability (F) of PQA-AZ4 and PQA-AZ6 after i.g. administration was calculated as:

(6) $$F\left(\%\right)=\frac{{\mathrm{AUC}}_{i.g.}}{{\mathrm{AUC}}_{i.v.}}\cdot \frac{D_{i.v.}}{D_{i.g.}}\cdot 100$$

where D_i.v._ and D_i.g._ are i.v. and i.g. doses of the studied molecules, respectively.

### 4.4. Behavioral Studies

#### 4.4.1. Forced Swim Test (FST)

The rats were injected for 21 consecutive days at a dose of 1 mg/kg, similar to the dose used in acute treatment, as per data presented in Supplementary Materials (Table S1). The FST was conducted according to the method of Porsolt [41]. On the first day of the experiment, the animals were gently placed individually for 15 min in Plexiglas cylinders (40 cm high, 18 cm in diameter) containing 15 cm of water maintained at 23–25 °C. On removal from water, the rats were placed for 30 min in a Plexiglas box under a 60 W bulb to dry. On the following day (24 h later), the rats were replaced in the cylinder and the total duration of immobility was recorded during the whole 5 min test period. Immobility was assigned when no additional activity was observed other than that necessary to keep the rat’s head above the water. Fresh water was used for each animal.

#### 4.4.2. Novel Object Recognition (NOR) Test

The rats were injected with the respective compounds for 21 consecutive days at the minimum active doses selected previously, based on acute treatment and assessment in the NOR test [10]. The protocol for the NOR test was adapted from the original work [23,42] and the test was performed according to the previously described protocol [10], 24 h after the last injection of the investigated compound. The discrimination index (DI) was calculated according to the formula:

(7) $$\mathrm{DI}=\frac{\left(\mathrm{EB}-\mathrm{EA}\right)}{\left(\mathrm{EA}+\mathrm{EB}\right)}$$

where EB is the exploration time of novel object during the T2 session and EA is the exploration time of familiar surroundings.

PQA-AZ4 and PQA-AZ-6 were administered for 60 and 30 min, respectively, before the T1 phase (the familiarization phase) [43]. MK-801 was used to attenuate learning and was administered at a dose of 0.1 mg/kg (i.p.) 30 min before the familiarization phase (T1). The total exploration time in T2 was used to determine the influence of the treatment on the exploratory activity of the animals.

#### 4.4.3. 18-Hour Spontaneous Activity Monitoring

Spontaneous activity was measured on the 20th day of the treatment with a special, innovative, radio-frequency identification system (RFID-system), TraffiCage (TSE-Systems, Bad Homburg, Germany). The test was conducted according to the method described previously [44,45]. The animals had RFID transmitters implanted subcutaneously, which tracked the time they spent in different areas of the cage.

#### 4.4.4. Body Weight Measurement

Body weight was measured every second day, immediately before the administration of drugs.

### 4.5. Ex Vivo Biochemical Studies

#### 4.5.1. Tissue Collection

Animals in all treatment groups were sacrificed by rapid decapitation immediately after the behavioral tests, when the brains and blood samples were collected. For quantitative reverse transcription-polymerase chain reaction (RT-qPCR), the brains of rats were rapidly removed, and the hippocampus and prefrontal cortex were dissected on an ice-cold glass plate. The tissues were frozen in dry ice and stored at −80 °C until required.

The blood samples were collected immediately after decapitation and then centrifuged at 600× *g* 915 min, 4 °C, to separate the plasma. The blood was used to determine metabolic parameters such as glucose, cholesterol, and triglyceride levels, together with liver enzymatic activity.

#### 4.5.2. Quantitative Real-Time PCR

The total RNA was extracted from tissues using an RNA Isolation kit (Thermo Fisher Scientific, Rockford, IL, USA), and its concentration was normalized to 20 ng/μL. Reverse transcription was performed with a High-Capacity Reverse Transcription Kit (Life Technologies, Grand Island, NY, USA). A qPCR fast 96-well reaction plate was used for the process with TaqMan Gene Expression assay (Life Technologies) for brain-derived neurotrophic factor *Bdnf* (Rn02531967_s1) on an Applied Biosystems^®^ 7500 Fast Real-Time PCR Instrument (Applied Biosystems, Foster City, CA, USA), according to the manufacturer’s protocol. Glyceraldehyde-3-phosphate dehydrogenase, *Gapdh* (Rn01775763_g1), was selected as endogenous control, based on the pilot experiment. Relative normalized expression was calculated using the ΔΔCq method.

#### 4.5.3. Metabolic Parameters

Standard enzymatic spectrophotometric tests (Biomaxima S.A., Lublin, Poland) were used to determine glucose, cholesterol, and triglyceride levels in the serum. In the presence of an appropriate enzyme, the substrate was converted to a colored compound. Absorbance was measured at a wavelength of 500 nm.

#### 4.5.4. Liver Enzymatic Activity

To determine alanine aminotransferase (AlaT), asparagine aminotransferase (AspaT), alkaline phosphatase (ALP), or gamma-glutamyl transpeptidase (γGTP) activities in the serum, a standard enzymatic spectrophotometric test (Biomaxima S.A., Lublin, Poland) was used. Absorbance was measured at a wavelength of 340 nm (AlaT, AspaT) or 405 nm (ALP, γGTP).

#### 4.5.5. Ferric-Reducing Antioxidant Power (FRAP) Assay

The assay was performed according to Benzie and Strain [46] with some modifications. The FRAP working solution was prepared before the start of the analysis, namely, 0.3 mol acetate buffer (pH 3.6), 0.01 mol TPTZ (2,4,6-tripyridyl-s-triazine; Sigma-Aldrich) in 0.04 mol HCl (POCh, Gliwice, Poland), and 0.02 M FeCl_3_×6H_2_O in water (iron (III) chloride hexahydrate; Chempur, Piekary Śląskie, Poland) were mixed in a volumetric ratio of 10:1:1 and protected from light.

Next, 20 µL of the plasma sample tested, brain homogenate, or FeSO_4_×7H_2_O solution was mixed with 180 µL of the FRAP working solution. The mixtures obtained were incubated at 37 °C for 30 min and their absorbance was measured at 593 nm. FeSO_4_×7H_2_O (100–1000 µM/L) was used for a calibration curve. Deionized water with FRAP solution was used as a blank.

### 4.6. Cytotoxic Effects of PQA-AZ4 and PQA-AZ-6 on Normal Human Dermal Fibroblast Cells

Alpha-MEM and the normal human dermal fibroblast cell line (NHDF) were obtained from Lonza (Lonza, Warsaw, Poland). Phosphate-buffered saline (PBS), fetal bovine serum (FBS), L-glutamine, and 100× antibiotic–antimycotic were from Cytogene (Zgierz, Poland). MTT (3-(4,5-dimethylthiazol-2-yl)-2,5-diphenyltetrazolium bromide) was from Sigma-Aldrich (Poznan, Poland) and DMSO was purchased from Archem (Kamieniec Wroclawski, Poland).

The viability of NHDF cells after treatment with PQA-AZ4 and PQA-AZ-6 was assayed using the previously described methodology. Briefly, 4.5 × 10^3^ cells/well were seeded into a 96-multiwell plate [47,48] and viability was evaluated using the MTT assay [49]. Different volumes of each compound dissolved in DMSO (final concentrations 4.68, 9.37, 18.75, 37.5, and 75 μM, respectively), as well as DMSO itself as solvent control, were added to each well, mixed, and incubated for 48 h. Finally, the optical density was measured at 560 nm with a reference wavelength of 670 nm using an Asys UVM 340 Microplate Reader (Cambridge, UK). Results were expressed as the percentage of survival cells concerning the control (not treated cells).

### 4.7. Data Analysis and Statistics

The behavioral and in vitro results were expressed as the means ± standard error of the mean (SEM), while the quantitative real-time PCR results were presented as the means ± standard division (SD). The data of FST and NOR were evaluated using one-way analysis of variance (one-way ANOVA) followed by a Bonferroni post hoc test. The results of biochemical studies (percent of tissue weight; glucose, triglyceride, and cholesterol levels; liver enzymatic activity; FRAP quantitative real-time PCR results assay) were compared using the Student’s *t*-test. Two-way analysis of variance (two-way ANOVA) followed by a Bonferroni post hoc test was used to evaluate changes in body weight and spontaneous activity. The cytotoxicity results were analyzed by one-way ANOVA with the post hoc Dunnett’s test for multiple comparisons. The normality of data sets was determined using the Shapiro–Wilk test. A value of *p* < 0.05 was considered statistically significant. Graph Pad Prism 6.0/7.0 and Statistica 13.1 were used for data analysis.

## Figures and Tables

**Table 1:** Structure and binding profile of compounds PQA-AZ4 and PQA-AZ6.

		PQA-AZ4	PQA-AZ6
Structure		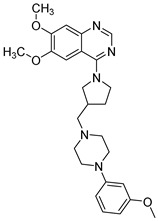	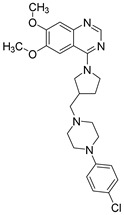
Chemical name		6,7-Dimethoxy-4-(3-((4-(3-methoxyphenyl)piperazin-1-yl)methyl)pyrrolidin-1-yl)quinazoline	4-(3-((4-(4-Chlorophenyl)piperazin-1-yl)methyl)pyrrolidin-1-yl)-6,7-dimethoxyquinazoline
Affinity	5-HT1AR 5-HT7R	K_i_ = 18.0 ± 1.8 nM	K_i_ = 346.0 ± 20.2 nM
		K_i_ = 234.0 ± 15.0 nM	K_i_ = 136.0 ± 15.0 nM
Inhibition	PDE10A	IC_50_ = 7.07 µM	IC_50_ = 5.68 µM

**Table 2:** Pharmacokinetic parameters and brain uptake for PQA-AZ4 after i.v. and i.g. administration at a dose of 0.5 and 2 mg/kg to male rats, respectively.

	i.v.		i.g.	
	Plasma	Brain	Plasma	Brain
C_0_ [ng/mL]	431.1	-	-	-
AUC_0_→_t_ [ng·min/mL] * [ng·min/g]	42,859	40,806	7103	40,314
t_0.5_ [min]	67.3	220	92	214
MRT [min]	57.5	186	82.6	215
V_ss_ [L/kg]	0.8	-	-	-
Cl [mL/min/kg]	10.8	-	-	-
t_max_ [min]	-	15	60	60
C_max_ [ng/mL]	-	149.1	46.7	71.4
F [%]	8.29			
Brain/Plasma ratio	0.9		5.68	

* Concentration in the brain; C_0_—initial concentration; AUC—area under the curve; t_0.5_—terminal half-life; V_ss_—the volume of distribution at steady state; Cl—systemic clearance; MRT—mean residence time; C_max_—maximum concentration; t_max_—time to reach C_max_; F—bioavailability.

**Table 3:** Pharmacokinetic parameters and brain uptake for PQA-AZ6 after i.v. and i.g. administration at a dose of 0.5 and 2 mg/kg to male rats, respectively.

	i.v.		i.g.	
	Plasma	Brain	Plasma	Brain
C_0_ [ng/mL]	101.6	-	-	-
AUC_0_→_t_ [ng·min/mL] * [ng·min/g]	5616	48,321	8619	45,623
t_0.5_ [min]	54.2	597	216.8	1928
MRT [min]	44.5	444	66.5	719
V_ss_ [L/kg]	4.8	-	-	-
Cl [mL/min/kg]	85.2	-	-	-
t_max_ [min]	-	15	15	15
C_max_ [ng/mL]	-	113.4	99.5	341.5
F [%]	38.4			
Brain/Plasma ratio	8.60		5.29	

* Concentration in the brain; C_0_—initial concentration; AUC—area under the curve; t_0.5—_terminal half-life; V_ss_—the volume of distribution at steady state; Cl—systemic clearance; MRT—mean residence time; C_max_—maximum concentration; t_max_—time to reach C_max_; F—bioavailability.

**Table 4:** The effect of repeated administration of compounds PQA-AZ4, PQA-AZ6, and PQ10 on the exploration activity of rats in the NOR test in (+)-MK-801 treated rats.

Treatment	Dose (mg/kg)	Total Exploratory Time in T2 Session (s)
Vehicle + vehicle	0 + 0	49.67 ± 4.63
(+)-MK-801 + vehicle	0.1 + 0	44.00 ± 4.05
PQA-AZ4 + (+)-MK-801	1 + 0.1	28.83 ± 2.50 *p* < 0.01 vs. veh; *p* < 0.05 vs. (+)-MK-801 F(2,16) = 7.3773; *p* < 0.01
Vehicle + vehicle	0 + 0	49.67 ± 4.63
(+)-MK-801 + vehicle	0.1 + 0	44.00 ± 4.05
PQA-AZ6 + (+)-MK-801	3 + 0.1	39.57 ± 4.39 F(2,17) = 1.3060; NS
Vehicle + vehicle	0 + 0	49.67 ± 4.63
(+)-MK-801 + vehicle	0.1 + 0	44.00 ± 4.05
PQ10 + (+)-MK-801	0.3 + 0.1	47.13 ± 5.69 F(2,18) = 0.3006; NS

Compounds PQA-AZ4, PQA-AZ6, and PQ-10 were administered i.p. once daily for 21 consecutive days. (+)-MK-801 was injected i.p. 30 min before the first trial (familiarization, T1) phase. Values represent the mean ± SEM of the total exploratory time of both objects during the 3 min test session (T2) compared to the respective vehicle group (one-way ANOVA followed by Bonferroni’s post hoc test); NS = nonsignificant. N = 6–8.

## Data Availability

The row data presented in this study are available on request from the corresponding author.

## Supplementary Materials

The following supporting information can be downloaded at: https://www.mdpi.com/article/10.3390/ijms231911452/s1.
