# Supplementary material for: The Antidepressant-like Activity, Effects on Recognition Memory Deficits, Bioavailability, and Safety after Chronic Administration of New Dual-Acting Small Compounds Targeting Neuropsychiatric Symptoms in Dementia
Source: Int J Mol Sci. 2022 Sep 28;23(19):11452. doi: 10.3390/ijms231911452 (PMC9569954; doi:10.3390/ijms231911452)
Supplement: Supplementary file 1 [file ijms-23-11452-s001.zip › ijms-1917162-supplementary.pdf]

## Supplementary Materials:

### 1. Methods in pharmacokinetic studies

**Instrumentation and operating conditions**

The LC/ESI-MS/MS experiments were performed on a UPLC ACQUITY H-Class PLUS/Xevo TQD (Waters Corporation, USA) triple-quadrupole mass spectrometer equipped with an electrospray (ESI) ionization interface. Data acquisition and processing were accomplished using MassLynx data collection and integration software.

**Chromatographic conditions**

Chromatographic separation was performed on an Acquity UPLC BEH C18 (1.7 μm, 2.1 x 50 mm, Waters, USA) column with the column temperature set at 50 ˚C. The mobile phase consisted of water with the addition of 0.1% formic acid (solvent A) and acetonitrile, with the addition of 0.1% formic acid (solvent B) in isocratic elution, set at a flow rate of 0.5 mL/min.

**Mass spectrometric conditions**

The mass spectrometer parameters were as follows: cone voltage: 86 kV; the temperature of the heated nebulizer: 500 °C, and collision energy: 66 kV. Mass spectra were acquired by SRM with precursor/predominant product ion transitions for the analytes. The mass spectral Q1→Q3 transitions monitored for PQA-AZ4, PQA-AZ6, and internal standard (IS) were m/z 464.1→272.1, m/z 468.6→271.9 and m/z 305→248, respectively. The peak widths of precursor and product ions were set to 0.7 full-width half-height. Quantification was done via peak area ratio.

**Sample pretreatment**

The plasma and brain sample pretreatment procedure involved acetonitrile precipitation. A 10 μL aliquot of the internal standard (IS, PH002437, Merck, Darmstadt, Germany) working solution (5 μg/mL) was added to 100 μL of the collected mice plasma or brain samples, which were then vortex-mixed for 10 seconds. Thereafter, 200 μL of acetonitrile was added, the sample vortexed for a further 20 min, and then centrifuged at 10,000 rpm, for 10 min. The supernatant (200 μL) was then transferred to an insert and placed in an autosampler vial, and a 10 μL volume of this was injected onto the LC column.

Brain samples were thawed before use. Individual whole brains were weighed and placed in a glass mortar and pestle tissue grinder and homogenized with an appropriate amount of phosphate buffer (pH 7.4) in a 1:5 ratio. Afterward, 100 μL of tissue homogenates was transferred to new Eppendorf tubes and spiked with 10 μL of the internal standard working solution. All samples were stored on ice during the preparation process and followed by procedures similar to those described above.

### 2. Results

**Pharmacokinetic studies**

The pharmacokinetic profiles of PQA-AZ4 and PQA-AZ6 were determined after i.v. and i.g. administration to male rats at a dose of 0.5 mg/kg and 2 mg/kg, respectively. The concentration of target compounds in plasma and brain was determined using an LC/ESI-MS/MS system.

The plots of mean plasma and brain concentrations versus time profile for the investigated compounds after i.v. and i.g. administration are depicted in Figures S1–S8, respectively.

C [ng/mL]

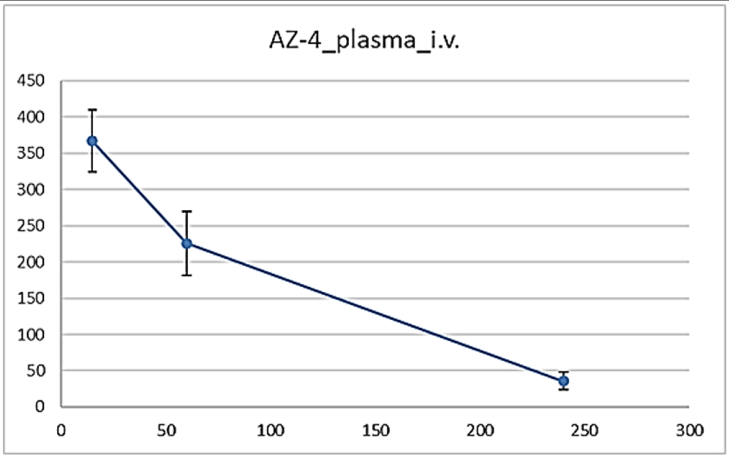


t [min]

**Figure S1.** Concentration–time profile for PQA-AZ4 in plasma after i.v. administration to rats at a dose of 0.5 mg/kg.

C [ng/mL]

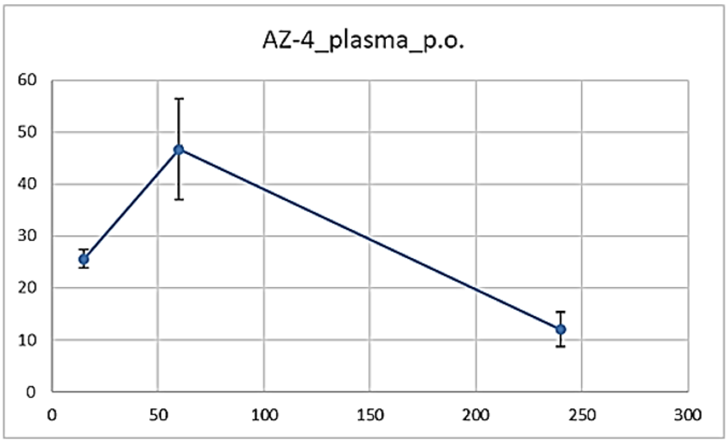


t [min]

**Figure S2.** Concentration–time profile for PQA-AZ4 in plasma after i.g. administration to rats at a dose of 2 mg/kg.

C [ng/mL]

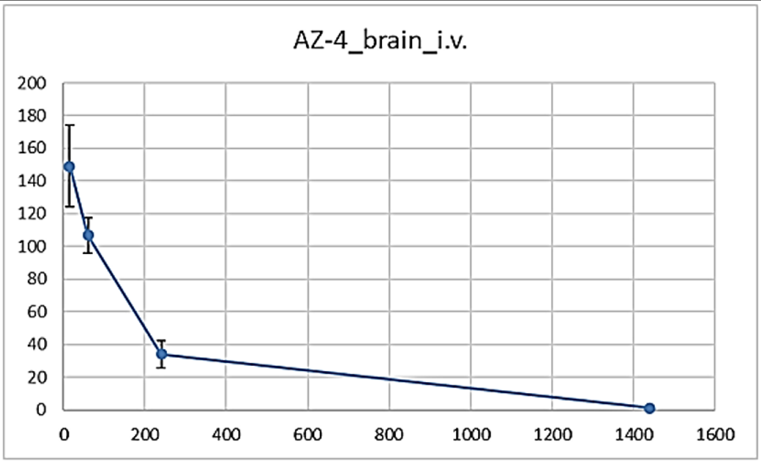


t [min]

**Figure S3.** Concentration–time profile for PQA-AZ4 in the brain after i.v. administration to rats at a dose of 0.5 mg/kg.

C [ng/mL]

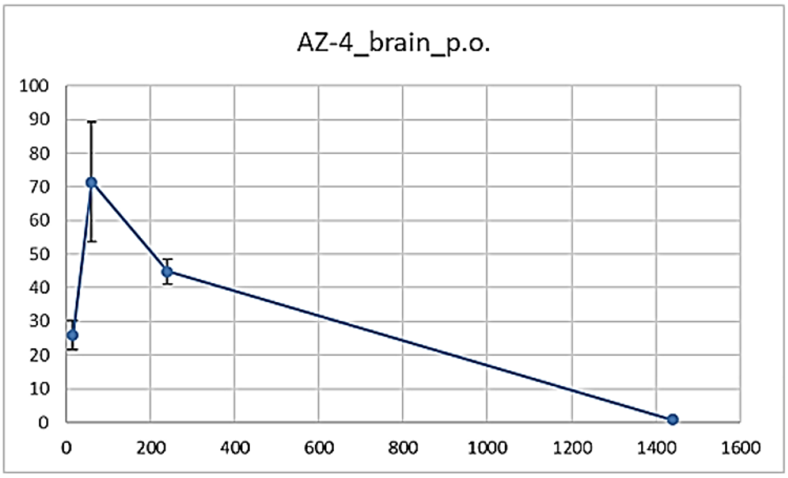


t [min]

**Figure S4.** Concentration–time profile for PQA-AZ4 in the brain after i.g. administration to rats at a dose of 2 mg/kg.

C [ng/mL]

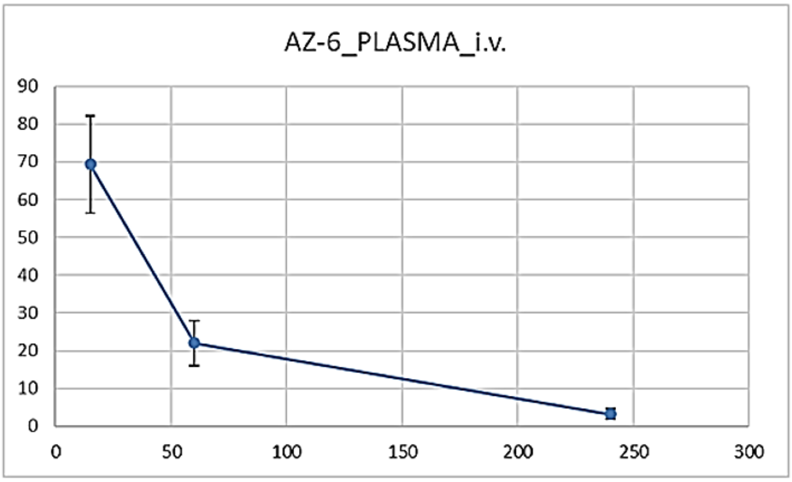


t [min]

**Figure S5.** Concentration–time profile for PQA-AZ6 in plasma after i.v. administration to rats at a dose of 0.5 mg/kg.

C [ng/mL]

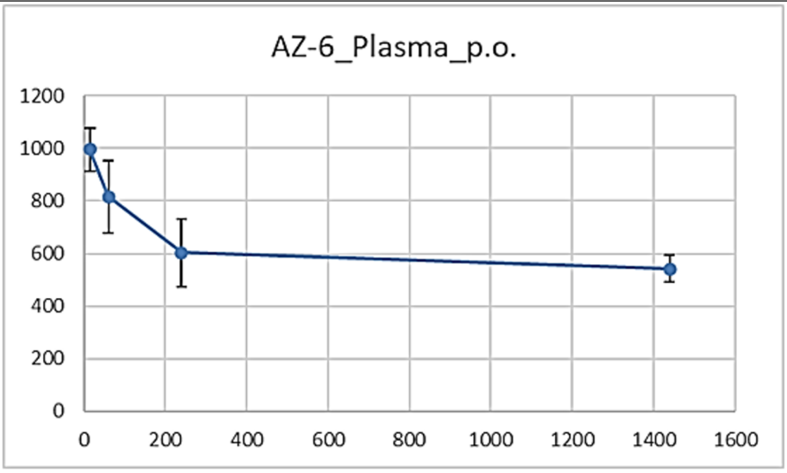


t [min]

**Figure S6.** Concentration–time profile for PQA-AZ6 in plasma after i.g. administration to rats at a dose of 2 mg/kg.

C [ng/mL]

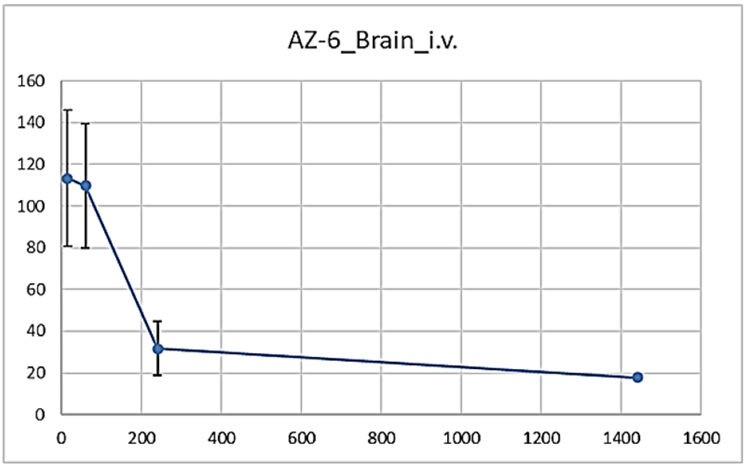


t [min]

**Figure S7.** Concentration–time profile for PQA-AZ6 in brain after i.v. administration to rats at a dose of 0.5 mg/kg.

C [ng/mL]

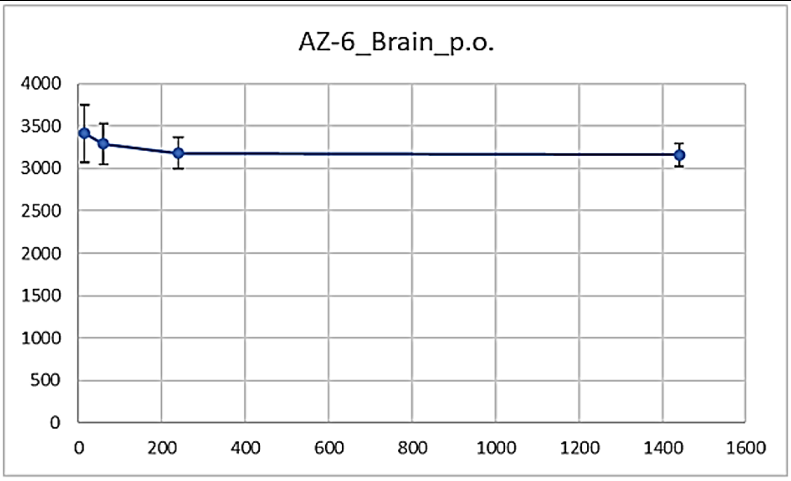


t [min]

**Figure S8.** Concentration–time profile for PQA-AZ6 in the brain after i.g. administration to rats at a dose of 2 mg/kg.

**Behavioral studies**

**FST after acute administration of the investigated compounds**

Rats were injected 60 min before the test at a dose of 1 mg/kg. FST was carried out according to the method of Porsolt [45], and the same procedure was used for chronic treatment.

Table S1. Effect of acute treatment of PQA-AZ4, PQA-AZ6, and PQ-10 on the immobility time in the FST in rats.

| Compound | Dose (mg/kg) | Immobility time (s) |
|---|---|---|
| vehicle | 0 | 255.9 ± 8.6 |
| PQA-AZ4 | 1 | 244.8 ± 8.9<br>$F(1,14)=0.8131$; NS |
| vehicle | 0 | 255.9 ± 8.6 |
| PQA-AZ6 | 1 | 255.4 ± 7.0<br>$F(1,14)=0.0020$; NS |
| vehicle | 0 | 255.9 ± 8.6 |
| PQ-10 | 1 | 249.1 ± 10.4<br>$F(1,14)=0.2493$; NS |

Compounds were injected i.p. 60 min before the test. Values represent the means ± SEM of immobility in the FST during a 5 min test session. The data were analyzed using one-way ANOVA, followed by Bonferroni's post hoc test; NS = nonsignificant; N=7-8.
